# Molecular mechanisms of *Eda*‐mediated adaptation to freshwater in threespine stickleback

**DOI:** 10.1111/mec.16989

**Published:** 2023-05-16

**Authors:** Carlos E. Rodríguez‐Ramírez, Melanie Hiltbrunner, Verena Saladin, Stephanie Walker, Araxi Urrutia, Catherine L. Peichel

**Affiliations:** ^1^ Division of Evolutionary Ecology, Institute of Ecology and Evolution University of Bern Bern Switzerland; ^2^ Department of Biology and Biochemistry, and Milner Centre for Evolution University of Bath Bath UK; ^3^ Institute of Ecology UNAM Mexico City Mexico

**Keywords:** alternative splicing, *Eda*, genetics of adaptation, lateral line, lateral plates, threespine stickleback

## Abstract

A main goal of evolutionary biology is to understand the genetic basis of adaptive evolution. Although the genes that underlie some adaptive phenotypes are now known, the molecular pathways and regulatory mechanisms mediating the phenotypic effects of those genes often remain a black box. Unveiling this black box is necessary to fully understand the genetic basis of adaptive phenotypes, and to understand why particular genes might be used during phenotypic evolution. Here, we investigated which genes and regulatory mechanisms are mediating the phenotypic effects of the *Eda* haplotype, a locus responsible for the loss of lateral plates and changes in the sensory lateral line of freshwater threespine stickleback (*Gasterosteus aculeatus*) populations. Using a combination of RNAseq and a cross design that isolated the *Eda* haplotype on a fixed genomic background, we found that the *Eda* haplotype affects both gene expression and alternative splicing of genes related to bone development, neuronal development and immunity. These include genes in conserved pathways, like the BMP, netrin and bradykinin signalling pathways, known to play a role in these biological processes. Furthermore, we found that differentially expressed and differentially spliced genes had different levels of connectivity and expression, suggesting that these factors might influence which regulatory mechanisms are used during phenotypic evolution. Taken together, these results provide a better understanding of the mechanisms mediating the effects of an important adaptive locus in stickleback and suggest that alternative splicing could be an important regulatory mechanism mediating adaptive phenotypes.

## 1 INTRODUCTION

Understanding the connection between genetic variation and adaptive phenotypic variation is one of the main goals in evolutionary genetics. It is a challenging task, but in recent years the genes that underlie adaptive traits have been identified in some systems (Bomblies & Peichel, 2022). For example, a difference in coat colour between deer mice (*Peromyscus maniculatus*) on different soils is controlled by *Agouti* (Linnen et al., 2009); loss of defensive lateral plates in freshwater threespine stickleback (*Gasterosteus aculeatus*) is controlled by *Eda* (Colosimo et al., 2005); industrial melanization of the peppered moth (*Biston betularia*) was caused by the insertion of a transposable element in the first intron of *cortex* (Hof et al., 2016); and pollinator‐specific flower colour in two sister species of monkeyflowers (*Mimulus lewisii* and *Mimulus cardinalis*) is controlled by *LAR1* (Yuan et al., 2016). However, even when a specific adaptive locus has been identified, the specific regulatory mechanisms and downstream molecular pathways mediating its effects on phenotypic variation often remain unknown (Bomblies & Peichel, 2022). A better understanding of how the genetic changes in adaptive loci impact the interactions of these genes in regulatory networks might explain why certain genes and molecular pathways tend to be re‐used in the evolution of certain phenotypes instead of functionally similar alternatives (Stern, 2013).

While most studies have focused on changes in gene expression as a mechanism underlying phenotypic evolution, a growing body of evidence suggests that alternative splicing might also be important for adaptation and phenotypic evolution (Bush et al., 2017; Chen et al., 2012; Singh & Ahi, 2022; Verta & Jacobs, 2022; Wright et al., 2022). Alternative splicing (AS) regulates which exons and/or introns from a gene are retained in the mature messenger RNA (mRNA), allowing different mRNA isoforms and proteins to be coded from the same gene, thereby increasing proteomic diversity. AS has been found in animals, plants and fungi (Bush et al., 2017; Chaudhary et al., 2019; Singh & Ahi, 2022; Wright et al., 2022). Between 92 and 95% of the genes in the human genome are estimated to undergo alternative splicing (Pan et al., 2008; Wang et al., 2008). Types of AS include exon skipping, exon shuffling, intron retention and use of alternative 5′ and 3′ splice sites. Exon skipping is the most common type in animals, while intron retention is more common in plants (Kim et al., 2007; Marquez et al., 2012; Wang & Brendel, 2006). Several recent studies have found evidence for a role of AS in both phenotypic evolution and adaptation. For example, a mutation affecting splicing in *Msx2a* contributes to reduction in dorsal spine length, a trait involved in defence against predators, in freshwater threespine stickleback populations (Howes et al., 2017), and upregulation of an *Agouti* splice isoform is involved in the evolution of cryptic coat coloration in two species of deer mice (Mallarino et al., 2017). Genome‐wide transcriptomic analyses have revealed changes in splicing between genetically similar but phenotypically distinct head and body lice ecotypes (Tovar‐Corona et al., 2015), between jaws from cichlid species occupying different trophic niches (Singh et al., 2017) and between benthic and pelagic ecotypes of Arctic charr (Jacobs & Elmer, 2021). These data point to the potential for AS to underlie adaptive phenotypic variation; however, the relative contribution of AS to adaptive phenotypic variation in comparison with differential gene expression is not well understood.

Threespine stickleback (*G. aculeatus*) are a great model to study the genetic and molecular mechanisms of adaptation. After the Last Glacial Maximum, approximately 15,000 years ago, individuals from marine populations in the Northern hemisphere independently colonized newly formed freshwater environments, resulting in the repeated evolution of phenotypic differences between marine and freshwater sticklebacks (Bell & Foster, 1994). This independent and replicated adaptation to freshwater makes threespine stickleback a very powerful system to study questions related to adaptation, phenotypic evolution and the repeatability of evolution (Peichel & Marques, 2017). One well‐studied trait is the repeated loss of bony lateral plates in most freshwater populations. These bony plates are known to provide protection against bird and fish predation in clear and open‐water environments, such as the ocean or large lakes (Kitano et al., 2008; Leinonen et al., 2011; Reimchen, 1992, 2000; Reimchen et al., 2013). Several studies have documented rapid and strong selection for the loss of lateral plates in freshwater (Barrett et al., 2008; Bell & Aguirre, 2013; Bell et al., 2004; Gelmond et al., 2009; Rennison et al., 2015; Rouzic et al., 2011; Schluter et al., 2021) although the selective pressure driving this lateral plate reduction is still not clear (Archambeault, Durston, et al., 2020). Gene mapping and transgenic studies have shown that *Ectodysplasin A* (*Eda*) is the main gene controlling this phenotype (Colosimo et al., 2004, 2005). *Eda* signalling is known to affect the development of ectodermal appendages like hair, teeth, feathers and scales in vertebrates, (Cui & Schlessinger, 2006; Sadier et al., 2014). In threespine stickleback, *Eda* also has pleiotropic effects on the patterning of the sensory neuromasts that make up the lateral line (Archambeault, Bärtschi, et al., 2020; Mills et al., 2014; Wark et al., 2012) and in schooling behaviour (Greenwood et al., 2013, 2016). In the threespine stickleback genome, *Eda* is in a 16 kb haplotype on chromosome IV that contains fixed genetic differences between marine and freshwater populations (Archambeault, Bärtschi, et al., 2020; Colosimo et al., 2005; Jones et al., 2012; O'Brown et al., 2015). Individuals that have two marine alleles (hereafter called C) of this haplotype are completely plated, while individuals that have two freshwater alleles (hereafter called L) are low‐plated. In the Puget Sound population used for this study, fish that are heterozygous for *Eda* are completely plated (Archambeault, Bärtschi, et al., 2020), but this is not the case in all populations (Colosimo et al., 2004; Laurentino et al., 2022). The haplotype also includes two other genes, *tumour necrosis factor superfamily member 13b* (*Tnfsf13b*) and *glycoprotein A‐rich protein* (*Garp*). Both genes have immune functions in humans; *Tnfsf13b* codes for a cytokine (BAFF) that is important for B cell survival and homeostasis (Schweighoffer & Tybulewicz, 2018; Smulski & Eibel, 2018), while *Garp* codes for a transmembrane receptor protein that regulates the function of regulatory T‐cells (Metelli et al., 2018). It is still unclear whether these two genes play a role in freshwater adaptation in threespine stickleback, by for example, mediating immune differences between the ecotypes, or if they are just tightly linked with *Eda* in the haplotype. There is some evidence for an effect of the *Eda* haplotypes in the expression of target immune genes in F2 individuals derived from marine and freshwater crosses (Robertson et al., 2017), which raises the possibility of an adaptive role of these two genes. However, this study did not have the resolution to distinguish between the effects of the *Eda* haplotype and linked genes.

Despite our knowledge of the link between the *Eda* genotype and several phenotypes, we still have little knowledge of the downstream molecular mechanisms by which the *Eda* haplotype mediates its known phenotypic effects or whether there are other phenotypic effects of the haplotype. To address these questions, here we compare the transcriptomes of threespine stickleback siblings that possess the three different genotypes (CC, CL and LL) at the 16 kb *Eda* haplotype but otherwise share the same genomic background. We compared these individuals across two tissues: skin, where the lateral line and lateral plates develop; and head kidney which is a primary hemopoietic organ in bony fish similar to the bone marrow in mammals (Soulliere & Dixon, 2017). Specifically, we asked three main questions: (1) what is the effect of the *Eda* haplotype on differential gene expression and alternative splicing?; (2) can we identify candidate genes and pathways that mediate the known phenotypic effects of *Eda*? and (3) does the *Eda* haplotype change the expression and/or splicing of other genes and pathways that might mediate other, previously unknown, phenotypic effects?

## 2 MATERIALS AND METHODS

### 2.1 Ethics statement

Animal husbandry and experimental procedures were approved by the Veterinary Service of the Department of Agriculture and Nature of the Canton of Bern (VTHa# BE4/16 and BE82/17).

### 2.2 Fish cross design and care

**FIGURE 1:**
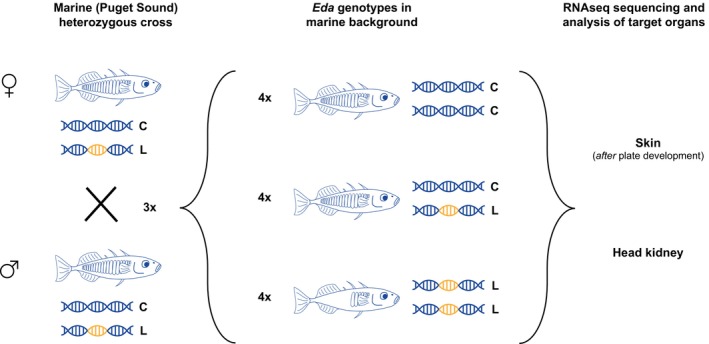
Experimental design. Marine sticklebacks that are heterozygous for the *Eda* haplotype have a completely plated phenotype and a marine genomic background (blue) but carry one copy of the completely plated C haplotype (blue) and one copy of the low‐plated L haplotype (yellow). Crossing these individuals results in offspring with the three *Eda* genotypes (CC, CL and LL) on the same marine genomic background. RNA from the skin and head kidney of these individuals was sequenced to test for the effect of the different *Eda* haplotypes on the transcriptome.

To quantify the effects of the *Eda* haplotype on the transcriptome, we crossed marine threespine stickleback that were heterozygous for the *Eda* haplotype. This cross design provided fish with the same genomic background that varied only on their *Eda* genotype, thus disentangling the effect of the *Eda* haplotype from the rest of the genome (Figure 1). The individuals used in this study were F3 descendants of heterozygous wild fish collected in Puget Sound, WA, USA in the summers of 2015 and 2016 as previously described (Archambeault, Bärtschi, et al., 2020; Archambeault, Durston, et al., 2020). We generated these F3 individuals by making three independent crosses (families A, B and C) between F2 females and males that were heterozygous for the *Eda* haplotype. The resulting F3 fish were raised at approximately 15.0°C in near freshwater conditions of 3.5 parts per thousand (ppt) Instant Ocean salt (Aquarium Systems, Sarrebourg, France). Fish were fed brine shrimp nauplii twice a day, except for weekends when they were fed only once a day. They were exposed to a light cycle of 11 h of daylight (3450 lumens), 1 h of sunset, 11 h of moonlight (600 lumens) and 1 h of sunrise. When the F3 fish were between 129 day and 131 days post fertilization, two males and two females per *Eda* genotype (CC, CL or LL) from each of the three families (for a total of 36 individuals) were sacrificed in MS‐222, skin and head kidney were dissected, and RNA was extracted for subsequent RNA sequencing (Figure 1).

### 2.3 DNA extractions and genotyping

DNA was extracted from fin tissue using a modified HotSHOT DNA extraction method as described (Archambeault, Bärtschi, et al., 2020; Archambeault, Durston, et al., 2020). Parents of the F3 crosses were genotyped at several markers in the *Eda* haplotype listed in Table S1 to confirm they had the full 16 kb *Eda* haplotype. The F3 individuals were genotyped at *Stn382* to identify their *Eda* genotype and at *LRR* to identify their sex (Table S1).

### 2.4 Dissections, RNA extraction and sequencing

We dissected skin and head kidney from 36 individuals for RNA sequencing. Skin was dissected from both sides of the posterior flank of the fish (starting at the level of the third spine, until the end of the dorsal fin), which is the region where LL sticklebacks do not have lateral plates and CL and CC sticklebacks do. RNA was extracted using an Invitrogen TRIzol kit (Invitrogen, Basel, Switzerland) according to the manufacturer's instructions. RNA concentration was measured for each sample using the Qubit RNA B Assay Kit (Invitrogen), and RNA quality was determined on a Fragment Analyzer CE12 (Advanced Analytics, Agilent, Santa Clara, CA, USA). The Next Generation Sequencing Platform of the University of Bern prepared the TruSeq Stranded mRNA library preparation for each of the 72 samples (36 skin, 36 head kidney) and performed the paired‐end sequencing of the 72 libraries with 300 cycles on an Illumina NovaSeq 6000 S2 flow cell.

### 2.5 RNA‐seq data pre‐processing

The quality of the RNAseq reads was verified with *FastQC* v0.11.9 (https://www.bioinformatics.babraham.ac.uk/projects/fastqc/). We mapped the reads to the threespine stickleback reference genome v5 (Nath et al., 2021), using *STAR* v2.7.3a (Dobin et al., 2013) following the parameters previously used for threespine stickleback (Verta & Jones, 2019): ‐*outFilterIntronMotifs: RemoveNoncanonicalUnannotated*; ‐*chimSegmentMin* 50; ‐*alignSJDBoverhangMin* 1; ‐*alignIntronMin* 20; ‐*alignIntronMax* 200,000; ‐‐*alignMatesGapMax* 200,000 and ‐‐*limitSjdbInsertNsj* 2,000,000. However, we did not run STAR in the 2pass mode, because it increased our multimapping read rate by 5% and we did not benefit from the de novo splice junction identification since our downstream analysis focused on annotated genomic features. Next, we used *FeatureCounts* v2.0.1 (Liao et al., 2014) to count how many reads mapped to each genomic feature. We did this at two different levels: gene and exon. We ran featureCounts in paired‐ended mode (−p), allowing only for reversely stranded alignments (−s 2), as per the characteristics of our read libraries and excluding read pairs where one of the mates did not map (‐B) or if they mapped into a different strand or chromosome (‐C). We used MultiQC v1.8 (Ewels et al., 2016) to summarize the quality reports for all samples from FastQC, STAR and featureCounts. One skin sample from a heterozygous (CL) female from family C was removed from all further analysis because it had high multimapping rates in STAR (35.7%). All computationally intensive calculations were performed on the University of Bern HPC cluster UBELIX (http://www.id.unibe.ch/hpc).

### 2.6 Identification of differentially expressed genes (DEGs)

For the differential expression analysis, we used R v3.6.1 (R Core Team, 2019) and *edgeR* v3.26.8 (Robinson et al., 2010) available at the Bioconductor website (http://bioconductor.org). We used the gene‐level read counts we obtained from *featureCounts* as input and started by filtering lowly expressed genes, that is, genes with fewer than 10 read counts in 11 or more of the 35 (skin) or 12 or more of the 36 (head kidney) samples analysed for a given tissue. Next, we calculated library normalization factors for all samples and estimated gene expression dispersions using a weighted likelihood Empirical Bayes approach. Then, we used the *plotMDS()* function of the *limma* v3.40.6 R package (Ritchie et al., 2015) to run a modified multidimensional scaling (MDS) analysis which calculates the distance between each pair of samples based on the 500 top genes with the highest gene expression fold‐changes between that pair of samples. Afterwards, we fitted all data to a negative binomial generalized linear model (GLM) model using genotype as the main explanatory variable and controlling for family and sex effects. Finally, we used a quasi‐likelihood F‐test to identify differentially expressed genes (DEGs) between genotypes. Instead of testing for fold‐change differences from zero between our genotypes, we tested for differential expression relative to a minimum fold‐change threshold using the *edgeR* implementation of the TREAT method (McCarthy & Smyth, 2009). We focused on genes with a significantly higher than 0.585 log2 fold‐change (approximately a 1.5 fold‐change in gene expression) between genotypes. We set the *p*‐value cut‐off to 0.05 and performed correction for multiple testing with the false discovery rate (FDR) method (Benjamini & Hochberg, 1995).

### 2.7 Identification of differentially spliced genes (DSGs)

One method to identify differential splicing is to test genes for differential exon usage. This is based on the principle that when the splicing pattern of a gene changes, the relative expression of the exons of that gene also changes. Though it cannot identify all types of AS events, this method can identify exon skipping and exon swapping events, which comprise approximately half of the AS events in humans (Chaudhary et al., 2019). We used *edgeR's* implementation of the differential exon usage test to identify genes with evidence of differential splicing. We used the exon‐level count data from *featureCounts* as the input, and applied the same filtering, variance estimation and GLM fitting steps to the data as we did for the gene‐level data for the differential expression analysis. We then used quasi‐likelihood *F*‐tests to identify differential exon usage using the two complementary methods in *edgeR*. The first method, called the ‘gene‐level’ method, uses the exon‐level test statistics to obtain a gene‐level p‐value, while the second method, called the Simes' method (Simes, 1986), first calculates exon‐level p‐values and converts them into a single gene‐level p‐value. The ‘gene‐level’ method is better at detecting genes with several differentially spliced exons while the second method is better at identifying genes with only a minority of differentially spliced exons (Chen et al., 2008). Any gene found to have significant differential exon usage by either one or both methods was reported as a putatively differentially spliced gene (DSG).

### 2.8 Gene co‐expression analysis

To identify putative gene interaction networks in the skin and head kidney transcriptomes, we did a weighted gene co‐expression network analysis (Zhang & Horvath, 2005) using the R package *WGCNA v1.69* (Langfelder & Horvath, 2008). This analysis uses pairwise gene expression correlations across the transcriptome to infer how connected genes are to each other and to identify clusters of co‐expressed genes (modules) whose gene expression is highly correlated and therefore expected to be working together in the same biological processes. We used *featureCounts* count data filtered by *edgeR*'s gene expression filter as input for the analysis. Following *WGCNA* recommendations (https://horvath.genetics.ucla.edu/html/CoexpressionNetwork/Rpackages/WGCNA/index.html), we normalized and applied a variance stabilizing transformation on the count data using the *vst()* function of the *DESeq2 v1.24.0* R package (Love et al., 2014) and adjusted for the family effect using the *ComBat()* function from the *sva v3.32.1* R package (Leek et al., 2012). Using the normalized and adjusted data, we created a gene similarity matrix using the absolute value of the pairwise biweight midcorrelation between all genes in our dataset. Next, we calculated a weighted adjacency matrix from the similarity matrix by rising the latter to a power of β. This power of β is referred to as the soft threshold of the analysis because it is used to emphasize strong correlations in the weighted adjacency matrix and de‐emphasize weaker gene correlations. To calculate the appropriate value of the soft threshold for our data, we plotted the fit of our data to an approximate scale‐free topology model (Zhang & Horvath, 2005) for different values of β using the *WGCNA* function *pickSoftThreshold()*. The plot revealed a saturation of the scale‐free topology model fit for soft thresholds of 14 for the skin and 12 for the head kidney data (Figure S1). To enable cross‐tissue comparisons, we selected a conservative soft threshold of 14 for both tissues. Next, to further minimize the effect of noise and random correlations, we calculated a topological overlap matrix (TOM) from the adjacency matrix. The TOM was calculated by analysing not only the adjacency between a pair of genes, but also the overlap and similarity of their adjacency with other ‘third party’ genes. Finally, a hierarchical clustering algorithm was used to define the gene co‐expression modules. These steps were all performed by inputting the adjusted and normalized count data to the *blockwiseModules()* function of *WGCNA* with the following settings: *corType = “bicor”*, *maxPOutliers = 0.10*, *maxBlockSize = 18*,*000*, *TOMType = “signed”*, *power = 14*, *randomSeed = 1234*.

We also used *WGCNA* to obtain measures of network total connectivity (kTotal) for every gene in the skin and head kidney transcriptomes. The total connectivity of a gene is a measure of how co‐expressed that gene is with all other genes in the transcriptome, and it is calculated by summing the adjacency values of that gene with all other genes. Gene connectivity has previously been used as a proxy for pleiotropy (Featherstone & Broadie, 2002; Hämälä et al., 2020; Jacobs & Elmer, 2021; Rennison & Peichel, 2022; Wagner et al., 2007). We, therefore, used gene connectivity as a proxy to compare the levels of pleiotropy among three sets of genes: skin DEGs, skin DSGs and the complete skin transcriptome. For this analysis, we only included genes that were solely DEGs or DSGs, removing the six genes that were both DEGs and DSGs. We calculated the kTotal connectivity distribution of these sets of genes and then did pairwise comparisons of their medians. To test if the differences in the kTotal medians were significant, we used permutation tests. For each pairwise median kTotal comparison, we generated 10,000 random sets of genes with the same size as the sets of genes we were comparing and calculated the ratio of how many times the absolute difference in kTotal of the random permuted sets was the same or greater than the absolute differences in the real sets being compared. Using the same permutation approach, we similarly compared the medians of the distributions of average gene expression levels of skin DEGs‐only, skin DSGs‐only and the complete skin transcriptome. It was not possible to do this for the head kidney data due to the lack of DEGs and low number of DSGs in this tissue.

### 2.9 Gene ontology enrichment analysis

We did a gene ontology (GO) enrichment analysis to identify GO terms overrepresented in the DEGs, DSGs and co‐expressed gene modules in the g:GOst module of the g:Profiler webservice (Raudvere et al., 2019; Reimand et al., 2007) (https://biit.cs.ut.ee/gprofiler/gost). We selected the Ensembl stickleback annotation database for the analysis and used the list of genes that passed *edgeR's* gene expression filter as a background. All other settings were left on default. G:Profiler results also provided human phenotype (HP) annotations for stickleback, however, these were not included in the analysis as they did not add more information than the GO Terms. To summarize these results, we followed a published protocol (Reimand et al., 2019) to build a network of enriched GO Terms by gene overlap using the Enrichmap v3.3.2 app of Cytoscape v3.8.2 (Merico et al., 2010; Shannon et al., 2003). The resulting GO Term networks were given representative names based on the terms present in the network using the default settings of the AutoAnnotate v1.3.4 app of Cytoscape (Kucera, 2017).

### 2.10 Identifying putative immune functions of DEGs and DSGs

To determine whether the *Eda* haplotype might have an influence on immunity, we also manually looked up the function of every LL versus CC DEG in skin and all DSGs found in both tissues in NCBI Gene (https://www.ncbi.nlm.nih.gov/gene) or in Zfin (https://zfin.org/) and GeneCards (https://www.genecards.org/). When genes were identified as having immune functions in these databases, we looked for supporting literature. Genes with clear evidence of having important immune roles in other organisms were considered putative immune genes.

## 3 RESULTS

### 3.1 The *Eda* haplotype affects the expression and alternative splicing of hundreds of genes

**FIGURE 2:**
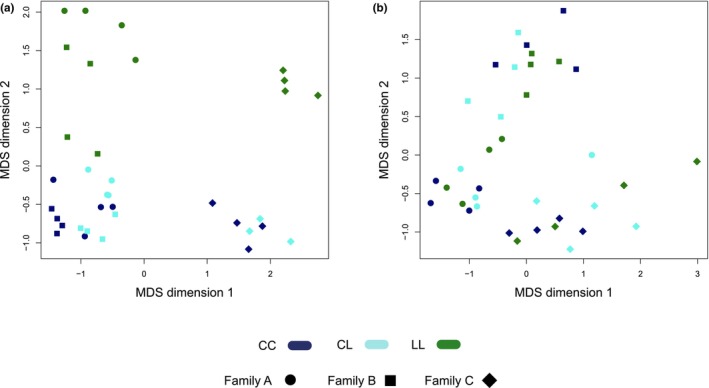
Samples cluster by *Eda* haplotype in skin but not head kidney. MDS plot of the pairwise distances between the gene expression profiles in (a) skin samples and (b) head kidney samples, based on the 500 genes with the largest pairwise changes in gene expression between each sample. Colour indicates the genotype of the samples: CC, dark blue; CL, light blue and LL, green, and the different shapes indicate the different families. In skin, the first MDS dimension separates one family (diamonds) from the other two (circles and squares). The second MDS dimension separates LL individuals from CC and CL individuals. In head kidney samples are clustered only by family.

**FIGURE 3:**
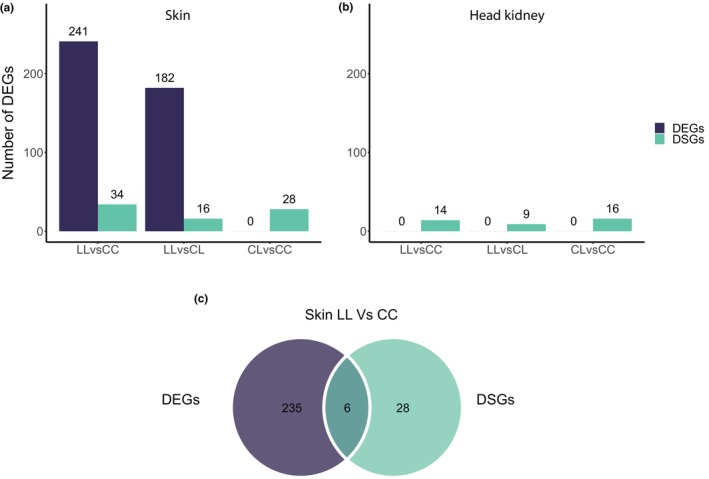
Differentially expressed genes (DEGs) and differentially spliced genes (DSGs) between *Eda* genotypes, in (a) skin and (b) head kidney. (c) Venn diagram of the overlap between DEGs and DSGs in the skin CC versus LL comparison.

Our results show an effect of the *Eda* haplotype in the skin and head kidney transcriptomes when controlling the genomic background, although the magnitude of this effect is quite different between the two tissues (Figures 2 and 3). In both tissues, an MDS analysis of the pairwise expression changes of all genes between samples separates individuals only by family (Figure S2). However, when focusing the analysis on the top 500 genes with the largest changes in gene expression between each pair of samples, the second dimension separated the LL *Eda* samples from the CC and CL *Eda* samples in skin (Figure 2a), mirroring the pattern of the plate phenotypes associated with these genotypes. This was not the case in head kidney, where the family effect was still the only factor driving the MDS (Figure 2b). We did not find any clustering of the samples by sex in the first two dimensions of either the skin or head kidney MDS (Figure S2). Consistent with the MDS results, we found no differentially expressed genes (DEGs) in head kidney and hundreds of DEGs in skin (Figure 3 and Table S2). There are dozens of differentially spliced genes (DSGs) between *Eda* genotypes in both skin and head kidney, but there are fewer DSGs in head kidney than in skin (Figure 3 and Table S2). *Eda* itself was a DEG in the skin CC versus LL comparison, but the other two genes of the haplotype, *Tnfsf13b* and *Garp* were not. More than half of the skin DEGs and DSGs between LL versus CL are also present in the LL versus CC comparison. The LL versus CC comparison captures approximately 94.1% of the DEGs and 59.3% of the DSGs in skin (Figure S3). Considering that CC and CL individuals have very similar lateral plate and lateral line phenotypes in this population (Archambeault, Bärtschi, et al., 2020), that the LL versus CC comparison allowed us to clearly distinguish the effect of the two *Eda* alleles, and that the effect of the *Eda* haplotype was stronger in the skin than in the head kidney transcriptome, we focused most of our downstream analysis on the LL versus CC comparison in the skin transcriptome.

### 3.2 Differentially expressed genes (DEGs) and differentially spliced genes (DSGs) in skin are mostly non‐overlapping

**FIGURE 4:**
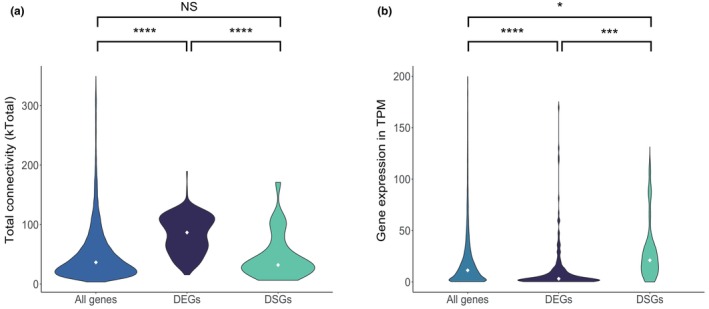
Skin DEGs have a greater gene co‐expression connectivity than skin DSGs, but DSGs are more highly expressed. (a) Violin plot showing the distribution of the values of total connectivity (kTotal) for all genes in the transcriptome, DSGs and DEGs. (b) Violin plot showing the distribution of the values of gene expression for all genes in the transcriptome, DSGs and DEGs. Expression values are normalized in transcripts per million (TPM). For visual clarity, 635 outliers with an expression value over 200 TPM in the ‘All genes’ category are not included in the plot. The six genes that were both DSGs and DEGs were not included in either analysis. In both plots, the white diamond in the middle represents the median of the distribution, and the results of permutation tests for each pairwise comparison are shown with asterisks (*p < .05; ***p < .001; ****p < .0001) or NS (non‐significant).

Of the 241 DEGs and 34 DSGs between the *Eda* CC and LL skin samples, only six were both differentially expressed and differentially spliced. Although this overlap is significant (*p* < .0001, 10,000 permutations), the low overlap suggests that these two regulatory mechanisms are mostly independent from each other (Figure 3c). It has been suggested that differential splicing might avoid constraints associated with differential expression of highly pleiotropic genes (Jacobs & Elmer, 2021; Rogers et al., 2021). To test whether pleiotropy could explain why some genes are regulated through gene expression and others through alternative splicing in our study, we compared gene co‐expression connectivity of DEGs and DSGs as a proxy for pleiotropy (see Section 2). We compared the connectivity distributions of the DEGs, DSGs and the transcriptome‐wide distribution and found that the DEGs have a higher total connectivity than the DSGs (DEGs median kTotal = 83.12, DSGs median kTotal = 46.22; *p* < .0001, 10,000 permutations) and that the DSGs had a total connectivity distribution not significantly different from the transcriptome‐wide distribution (transcriptome median kTotal = 36.39, DSGs median kTotal = 46.22; *p* = .2785, 10,000 permutations) (Figure 4a). Interestingly, we found the opposite pattern when comparing gene expression levels between the DEGs and the DSGs, with the DSGs more highly expressed than the DEGs (DSGs median = 21.38 TPM, DEG median = 3.07 TPM; *p* < .0001, 10,000 permutations) (Figure 4b). The DEGs also have a lower expression than the transcriptome‐wide median (DEGs median = 3.07 TPM, transcriptome median = 12.84 TPM; *p* < 1e−4, 10,000 permutations) while the DSGs have a higher expression (DSGs median = 21.38 TPM, transcriptome median = 12.84 TPM; *p* = .019, 10,000 permutations). These results suggest that factors like gene connectivity and expression level might be important in determining the type of regulatory mechanisms used to mediate phenotypic evolution.

To test whether DEGs and DSGs might be working in the same molecular pathways, we again used gene co‐expression analysis to identify modules of strongly co‐expressed genes. Of the 37 co‐expression modules we identified in skin (Figure S4), seven contain at least one DEG or DSG, and four of these contain both classes of genes (Table 1). Most of the DEGs (including *Eda*) are in a single co‐expression module (module M5), and a smaller cluster of 12 DEGs is in another module (module M27). Only seven out of 241 DEGs were not present in any co‐expressed module. By contrast, most (21 out of 34) of the DSGs were not in any of the co‐expression modules (Table 1). Five out of the six genes that were both DSGs and DEGs were found with most of the DEGs in module M5. These results suggest that most of the DEGs we identified are strongly correlated in their gene expression in the skin transcriptome and thus might be working in the same or closely related molecular pathways. Genes that are both DSGs and DEGs are correlated with other DEGs and might be interacting with them; however, most of the DSGs have independent patterns of expression and might have more indirect interactions with the DEGs and each other (Table 1).

**TABLE 1:** Distribution of the skin DEGs and DSGs across M0, which represents genes not belonging to any co‐expression module, and the seven out of 37 co‐expression modules containing at least one of these categories of genes in the skin transcriptome. Details of all modules are provided Figure S4 and Table S8.

Module	M0	M2	M5	M6	M7	M10	M27	M32
Only DSGs	20	3	1	1	1	1	0	1
Only DEGs	6	2	210	2	3	0	12	0
DEG and DSG	1	0	5	0	0	0	0	0
Total genes	10,791	1009	385	267	234	151	39	29

### 3.3 The *Eda* haplotype affects genes involved in bone development, neuronal development and immune response

**FIGURE 5:**
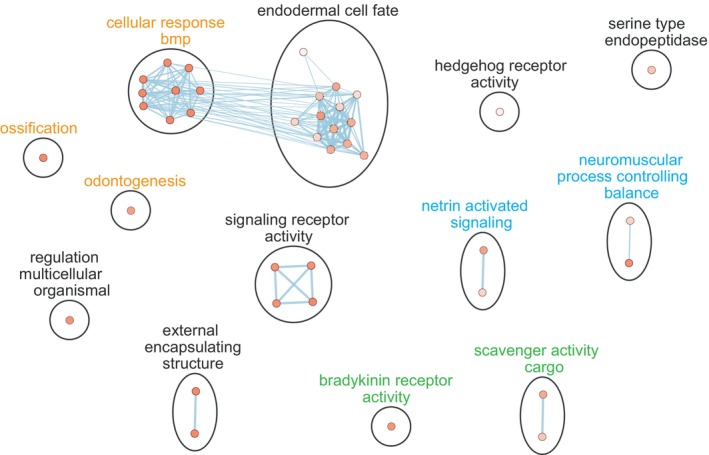
Summary networks of enriched GO terms in DEGs between *Eda* CC and LL individuals in skin. Nodes represent individual GO terms that were found to be significantly enriched in the 241 DEGs. Colour of the nodes represents the P‐value for the GO term. Blue lines represent gene similarity between GO terms. Black circles represent clusters of highly overlapping GO terms. Clusters labelled in orange have annotations related to bone development, clusters labelled in green have annotations related to immunity, clusters labelled in blue have annotations related to neuronal processes and clusters labelled in black have general annotations. Labels of clusters of annotations are based on WordClouds of the GO terms present inside the clusters. For clarity, general GO Terms present in more than 150 genes in the genome were not included in the network. For the full list of enriched GO Terms see Table S3.

Gene Ontology enrichment analysis revealed that the DEGs in skin are enriched in general development and signalling and in more specific processes like bone development (i.e. GO Terms like ‘ossification’, ‘odontogenesis’ and ‘BMP signalling’) and neuronal development (‘netrin receptor activity’ and ‘neuromuscular process controlling balance’) (Figure 5 and Table S3 for the full list of enriched GO Terms). There were no significantly enriched GO Terms for the DSGs, possibly because there were only 34 DSGs. However, more than half of the GO Terms present in DSGs are also present in the DEGs (51 out of 89) (Figure S5). Inspection of the individual GO annotations present in the DSGs revealed the presence of two genes with annotations related to cartilage development (C*ol11a2* and *Runx2b*) and three genes with neuronal annotations (*Cln3*, *Zc4h2* and *Anks1b*) (Table S4). Together, these results are consistent with the known effects of *Eda* on the lateral plates and sensory lateral line and suggest that the DEGs and DSGs underlying these GO terms are good candidates to be mediating these phenotypes.

**FIGURE 6:**
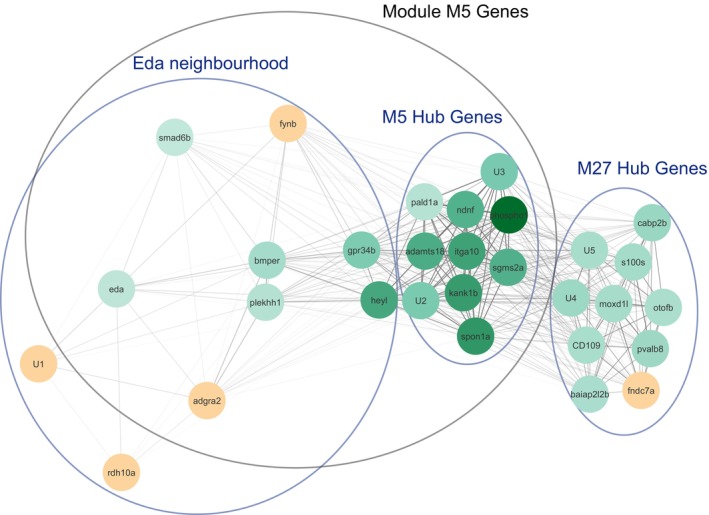
Gene co‐expression network of the top 10 genes co‐expressed with *Eda* (‘Eda neighbourhood’) and the 10 genes with the highest total connectivity in each of modules M5 and M27. Green circles indicate *Eda* LL versus CC DEGs, with darker shades indicating higher fold‐change between LL and CC individuals. Yellow circles indicate genes that are not significantly differentially expressed. Lines indicate co‐expression strength, with shorter and darker lines indicating stronger gene co‐expression between two genes. Gene *Rdh10a*, on the bottom left of the figure was manually brought closer to the rest of the genes for visualization purposes. Nodes with U1 to U5 labels are uncharacterized genes. Their Ensembl IDs are as follows: *ENSGACG00000017847* (U1), *ENSGACG00000016062* (U2), *ENSGACG00000008364* (U3), *ENSGACG00000015150* (U4) and *ENSGACG00000015153* (U5).

The gene co‐expression module M5 (where most of the DEGs are found) reveals similar GO enrichment results to the DEGs, except for the lack of the neuronal GO terms (Table S3). However, module M27 has the second‐most DEGs (Table 1) and has an enrichment of the ‘neuromuscular process controlling balance’ GO term found in the DEGs (Table S3). This module also has several genes annotated as being involved in lateral line development, vestibular reflex and sound perception, which are all systems that rely on hair cells (Table S4). Together, these results suggest that the effect of the *Eda* haplotype on the lateral plates seems to be represented mostly by co‐expression module M5, while the effect of the *Eda* haplotype on the patterning of the lateral line is represented by module M27. Interestingly, the gene co‐expression network of the 10 genes most closely co‐expressed with *Eda* (the *Eda* co‐expression neighbourhood) plus the top 10 connected genes (or hub genes) in modules M5 and M27 position M5 between *Eda* and module M27 (Figure 6). Since the distances in the network are based on how tightly genes are co‐expressed, which should correlate with how closely genes interact, the topology of the network suggests that the effect of the *Eda* haplotype on module M5 could be mediated by genes in the *Eda* co‐expression neighbourhood. These results further suggest that the effect of the *Eda* haplotype on module M27 could be mediated through the genes in module M5 (Figure 6). However, empirical studies manipulating the genes in these modules are necessary to test these hypotheses.

We also find evidence for a possible effect of the *Eda* haplotype on the immune response of skin and head kidney. The skin DEGs were enriched in GO terms for genes involved in the bradykinin pathway, which are strong inflammatory molecules, and in scavenger receptors which are involved in homeostasis and innate immunity (Alquraini & El Khoury, 2020; Canton et al., 2013) (Figure 5 and Table S3). One of the DSGs (*ENSGACG00000014601*) has an Ig‐like domain and a putative immune function‐related GO term (Table S4). Furthermore, a literature search of the skin DEGs revealed the presence of two genes with interesting links to immunity (Table S5). The *Ets1* (ETS proto‐oncogene 1) gene is involved in the development and/or function of T cells, B cells and natural killer cells (Dittmer, 2003; Garrett‐Sinha, 2013). The *Laptm4b* (lysosomal protein transmembrane 4 beta) gene regulates the immunosuppressor activity of regulatory T cells and is known to interact with *Garp*, one of the genes in the *Eda* haplotype (Huygens et al., 2015) (Table S5). Literature research also revealed an important immune gene, *Tbk1*, among the skin DSGs. *Tbk1* plays an important role mediating the interaction between multiple signalling pathways, many of which are related to immunity, namely pathogen detection, inflammation and immune response (Helgason et al., 2013).

In head kidney, the LL versus CC DSGs include genes annotated as involved in innate immune response, hemopoiesis, nervous system development and epidermal development (Table S4). Literature research revealed that the two genes with immune annotations, *itgb2* and *traf3*, do have important immune roles (Table S5). *Itgb2* (integrin, beta 2), also known as *lfa‐1* and *cd177*, is important for the function and migration of T cells, neutrophils and killer cells (Bai et al., 2017; Barber et al., 2004; Kristóf et al., 2013; Ostermann et al., 2002; Walling & Kim, 2018). *traf3* (TNF receptor‐associated factor 3) is a gene that plays an important role in antiviral innate immune response (Gao et al., 2021; Oganesyan et al., 2006; Tseng et al., 2010) and the regulation of B and T cells (Lin et al., 2015; Yi et al., 2015). Taken together, these results suggest the potential for a pleiotropic role of the *Eda* haplotype on immune function in the skin and head kidney.

### 3.4 The *Rmnd5b* gene is consistently differentially spliced in both skin and head kidney

To look for general effects of the *Eda* haplotype, we looked for genes that are consistently differentially expressed and/or differentially spliced in both skin and head kidney. There are no consistent DEGs between skin and head kidney, but there is one DSG, *Rmnd5b* (*required for meiotic nuclear division 5 homologue B*) (Table S2) which is a gene from chromosome IV located 500 kb downstream of *Eda. Rmnd5b* is a subunit of the GID/CTHL E3 ubiquitin ligase protein, which is involved in regulating cell proliferation and glucose metabolism (Lampert et al., 2018; Maitland et al., 2022; Santt et al., 2008). In both skin and head kidney, there is differential usage of exon 1 between *Eda* LL and *Eda* CC individuals (Figure S6). In head kidney, *Rmnd5b* is found in a co‐expression module mostly related to transcriptional regulation but that also includes genes related to myeloid cell homeostasis and erythrocyte differentiation (Tables S3 and S6). In skin, *Rmnd5b* is not part of any co‐expression module (Table S6). However, when we atomize the expression of *Rmnd5b* into its individual exons and analyse their co‐expression with the rest of the genes, we find exon 1 of *Rmnd5b* in module M5 together with *Eda* and most DEGs (Table S6). These results raise the possibility that differential splicing of *Rmnd5b* might mediate some of the *Eda* haplotype*'s* effects in skin and/or head kidney. The consistent effect on the splicing patterns of *Rmnd5b* suggests that there could be a cis‐regulatory effect from the *Eda* haplotype driving these differences. However, we also find two SNPs within exon 11 of *Rmnd5b* (positions 13320127 and 13320727) that are always homozygous for the reference allele in *Eda* CC individuals and heterozygous for the alternative allele in *Eda* LL individuals, such that one of the *Eda* haplotype L alleles is in linkage with the alternative allele at these SNPs (Table S7). Nonetheless, it is not clear how SNPs in exon 11 of *Rmnd5b* or in the *Eda* haplotype could be acting in cis to drive the consistent change in the splicing pattern of exon 1 between *Eda* CC and LL individuals because splicing regulatory regions tend to be found within the exon being regulated or in its adjacent introns (Lee & Rio, 2015; Lovci et al., 2013; Ule & Blencowe, 2019). Thus, further studies are necessary to verify whether and how the *Eda* haplotype affects the splicing of *Rmdn5b* and to test what role this might have in mediating its phenotypic effects.

## 4 DISCUSSION

In this study, we investigated the downstream effects of a 16 kb haplotype that has fixed differences between marine and freshwater threespine sticklebacks. This haplotype includes the gene *Eda* which is responsible for changes in lateral plates, lateral line and schooling behaviour between these ecotypes (Archambeault, Bärtschi, et al., 2020; Colosimo et al., 2004, 2005; Greenwood et al., 2016; Mills et al., 2014). We examined the effect of the *Eda* haplotype in the transcriptomes of skin and head kidney by isolating the three *Eda* genotypes (CC, CL and LL) on the same marine genomic background. There is a significant effect of the *Eda* haplotype on gene expression in skin, with hundreds of genes changing their expression by more than 1.5‐fold between genotypes. We also found that the phenotypic effects of the *Eda* haplotype might not only be mediated through changes in the gene expression but also through changes in alternative splicing (Figure 3a). Although differentially spliced genes (DSGs) are mostly non‐overlapping with differentially expressed genes (DEGs) (Figure 3b), several DSGs are involved in the same biological processes as DEGs. This suggests that both processes might be important to mediate the phenotypic effects of the *Eda* haplotype. The DEGs and DSGs in skin are related to skeletal tissue development and neuronal processes, making them good candidates for mediating the known effects of *Eda* on the number of lateral plates and the patterning of the lateral line. Furthermore, we found some evidence for a pleiotropic effect of the *Eda* haplotype in immunity, with genes related to inflammation and leucocyte function differentially expressed or spliced in skin and dozens of genes differentially spliced in head kidney, the main immune organ in fish.

### 4.1 The *Eda* haplotype affects gene expression and splicing in mostly different genes

To identify the most important mediators of the phenotypic effects of the *Eda* haplotype, we only considered genes with a more than 1.5‐fold difference in expression level between genotypes. That we found hundreds of DEGs in skin is a confirmation of the strong effect of this relatively small region of the genome (16 kb out of 450 Mb). Among these DEGs was *Eda* itself, which previously was found to have differences in expression levels between the C and L allele due to reduced responsiveness to *Wnt* signalling of the L allele (O'Brown et al., 2015). However, *Tnfsf13b* and *Garp*, the other two genes in the haplotype were not differentially expressed or spliced. The numbers of DEGs across the different genotype comparisons (LL vs. CC, CL vs. CC and LL vs. CL) mirror the phenotypic differences of these genotypes: there are no DEGs between the two completely plated genotypes (CC and CL) but overlapping and similar number of DEGs in the comparisons of the low‐plated genotype with the other two (LL vs. CC and LL vs. CL) (Figure 3a). This is consistent with the hypothesis that the DEGs we identified are involved in mediating the phenotypic effects of the *Eda* haplotype in skin.

To determine whether other regulatory mechanisms besides gene expression could be important in mediating the effects of *Eda*, we asked whether the *Eda* haplotype has an effect in alternative splicing, a regulatory mechanism that has recently been linked to phenotypic evolution and adaptation (Bush et al., 2017; Chen et al., 2012; Singh & Ahi, 2022; Verta & Jacobs, 2022; Wright et al., 2022). Though more limited than the effect on gene expression, we found that the *Eda* haplotype also affected alternative splicing of dozens of genes in skin. This is likely to be an underestimation of the number of DSGs, since the method that we used to identify DSGs, differential exon usage, is a conservative method that only detects changes in splicing involving complete exons. However, differential exon usage still accounts for roughly half of the splicing events in human (Chaudhary et al., 2019) and has the greatest potential for mediating modular changes in the protein function. We found that differential expression and differential splicing affect mostly different sets of genes, with only six genes being both differentially expressed and differentially spliced in the *Eda* CC versus LL comparison (Figure 3c). While a study comparing sympatric ecotypes of artic charr also found limited overlap between DEGs and DSGs (Jacobs & Elmer, 2021), a study comparing male and female transcriptomes of several bird species found almost half of the DSGs were also DEGs (Rogers et al., 2021) and a study in seasonal morphs of the *Bicyclus* butterfly found more than half of the DSGs were also DEGs (Steward et al., 2022).

In our study, DSGs and DEGs are mostly not found in the same gene co‐expression networks. While most DEGs are found together on module M5, most DSGs are not part of any co‐expression module (Table 1). This could be explained by the nature of the gene co‐expression analysis, which clusters genes with similar expression profiles, something that DEGs will tend to share, and the fact that it is a gene‐level analysis, so if a DSG has isoforms with different co‐expression profiles, they will be missed in the gene co‐expression analysis. This is supported by our results in the exon‐level co‐expression of *Rmnd5b*, where we find that the differentially spliced exon 1 is co‐expressed with module M5 (Table S6). However, despite these limitations of the gene co‐expression analysis, 13 DSGs are found in a gene co‐expression module, and 11 of those are found in modules that also include DEGs. These include five of the six DSGs that are also DEGs, which are found in module M5 together with most DEGs. So, while differential expression and differential splicing caused by the *Eda* haplotype tend to affect different groups of genes, some of the DEGs and DSGs are part of the same co‐expression networks and so might be working together to mediate the phenotypic effects of the haplotype.

### 4.2 Downstream effects of the *Eda* haplotype in skin include conserved and pleiotropic molecular pathways that are strong candidates to mediate the effects of *Eda* in skin

When comparing the transcriptomes of *Eda* CC and *Eda* LL individuals we find that genes with functions related to skeletal development (e.g. skeletal system development, ossification, odontogenesis, calcium ion binding) are more often differentially expressed than we would expect by chance (Figure 5 and Table S3). This result is consistent with the fact that these two genotypes underlie the two distinct lateral plate phenotypes in threespine sticklebacks (completely plated vs. low‐plated) and makes these DEGs strong candidates to be mediators of the effects of the haplotype in the bony lateral plates. One excellent candidate is the bone morphogenetic protein (BMP) pathway, which is a conserved pathway in animals that was first discovered for its role in bone formation (Salazar et al., 2016; Wang et al., 2014). However, this pathway is now known to have pleiotropic effects on tissue homeostasis, embryogenesis and development, including the development of ectodermal appendages (Cui & Schlessinger, 2006; Sadier et al., 2014; Wang et al., 2014). The BMP pathway and the *Eda* pathway have been found to regulate each other in mice (Sadier et al., 2014). In stickleback, a cis‐regulatory mutation in *Bmp6* is associated with an increased number of pharyngeal teeth in benthic populations (Cleves et al., 2014), while loss‐of‐function mutations in *Eda* result in loss of pharyngeal teeth (Wucherpfennig et al., 2019). Five of the skin DEGs we found are members of the BMP family, namely *Bmp2a*, *Bmp4*, *Bmp5*, *Bmp7a and Bmp8a*. *Bmp4* is particularly interesting because it has previously been connected to adaptive phenotypic changes in beak size in Darwin's finches (Abzhanov et al., 2004). Taken together, these results suggest that the BMP pathway is a strong candidate to be a mediator of the effect of *Eda* on the bony lateral plates.

However, the BMP pathway was not the only pathway present in the skin DEGs. Genes from the Hedgehog pathway, which also plays a role in the development of ectodermal appendages (Sadier et al., 2014), were also present more often than expected by chance among the DEGs (Figure 5 and Table S3). This includes the Indian hedgehog molecule a (*Ihha*), which has been shown to regulate BMP expression and bone formation (Rahman et al., 2015). Furthermore, though not statistically overrepresented in GO terms, we also find DEGs from other important signalling pathways like Wnt (*Lef1* and *Dkk1a*), Fgf (*Fgf13b* and *Fgfr4*) and Notch (*Dld and Egfl6*) (Table S3). Wnt and Fgf are known to also mediate the development of ectodermal appendages, including scale development in zebrafish (Aman et al., 2018; Cui & Schlessinger, 2006; Sadier et al., 2014). Furthermore, lower responsiveness to Wnt signalling has been connected to lower expression level of the freshwater *Eda* L allele (O'Brown et al., 2015). Among the DEGs from the Wnt pathway, we found the gene *Lef1*, a transcription factor which mediates Wnt activation of *Eda* expression in human cells (Durmowicz et al., 2002), and *Dkk1a* a Wnt antagonist (Table S2). The presence of a Wnt activator of *Eda* and an antagonist of Wnt among the skin DEGs suggests that there might be a negative feedback interaction between the *Eda* pathway and the Wnt pathway during the development of lateral plates in threespine stickleback, as found in mouse hair and in zebrafish scales (Aman et al., 2018; Cui & Schlessinger, 2006).

These signalling pathways are highly pleiotropic and are involved in much more than just bone and ectodermal appendage development. For example, the Wnt, Fgf and the Notch pathways are also involved in the development and patterning of the lateral line in zebrafish (Dalle Nogare & Chitnis, 2017; Kniss et al., 2016). The two DEGs from the Wnt pathway mentioned previously, *Lef1* and *Dkk1a*, are also involved in neuromast development (Table S4). Consistent with this, we found an enrichment of DEGs related to neuronal processes, namely including netrin activity and genes related to ‘neuromuscular process controlling balance’ (Figure 5). Netrins are a conserved family of diffusible proteins with chemotaxis characteristics, which are involved in axon guidance in the central nervous system (CNS). The enrichment of the ‘neuromuscular process controlling balance’ annotation was driven by three genes: cadherin‐related 23 (*Cdh23*), otoferlin b (*Otofb*) and calcium channel, voltage‐dependent, L type, alpha 1D subunit a (*Cacna1da*) (Table S3). These three genes are also annotated as being involved in sound perception, and *Cdh23* is also annotated as being involved in neuromast hair cell morphogenesis. These genes are interesting because the mammalian auditory and vestibular systems (the latter responsible for the sense of balance) and the fish lateral line system are all based on the use of hair cells to detect changes in balance, air and water pressure respectively (Mogdans, 2019; Roberts et al., 1988). As most functional annotations in stickleback are semi‐automatically imported from model organisms (including mammals like mouse or human) (Gaudet et al., 2011), it is not surprising that some of the genes involved in lateral line development would be annotated as involved in balance and sound perception. Interestingly, gene co‐expression module M27, which has 12 DEGs, is also enriched in genes with the ‘neuromuscular process controlling balance’ GO Term. This includes one DEG, *otofb* which affects the development of neuromast hair cells in zebrafish (Manchanda et al., 2021) and regulates the release of neurotransmitters in hair cells in humans (Roux et al., 2006; Yasunaga et al., 1999). Besides *Otofb*, module M27 also includes five other genes that are annotated as being involved in lateral line development, the auditory system, and/or the vestibular system (Table S4). Taken together, these results suggest that M27 might represent a network of genes involved in mediating the effect of the *Eda* haplotype on lateral line patterning. In addition, some of the genes in module M5, such as the DEGs connected to the netrin pathway, auditory system, balance and neuromast development, probably also contribute to this phenotype. This module also contains the BMP pathway, which is also important for the development and patterning of the central and peripheral nervous system (Gámez et al., 2013). For example, *Bmp4* limited the number of sensory neurons and the extent of terminal peripheral nerve innervation in mouse skin (Guha et al., 2004). Interestingly, the topology of the gene co‐expression network suggests that the influence of *Eda* on module M27 is mediated through module M5 (Figure 6). Taken together, these results suggest several DEGs in module M27 and M5 that are strong candidates for mediating the phenotypic effects of the *Eda* haplotype on the patterning of the lateral line.

Regarding the DSGs, we also found genes related to bone and neuronal development among the 34 DSGs between *Eda* LL and CC in skin. We did not find any significant enrichment of GO terms in the DSGs, but the GO enrichment analysis does not have much power with the relatively small number of DSGs. Thus, we looked at the GO Terms present in the DSGs and found three with annotations related to neuronal development (*Zc4h2*, *Cln3* and *Anks1b*), two genes related to cartilage development (*Runx2 and Col11a2*), and an uncharacterized gene on chromosome IV (*ENSGACG00000017917*), predicted by Uniprot to have cadherin domains and be involved in cell adhesion and calcium binding, both processes connected to bone development. Furthermore, while most DSGs were not found in any co‐expression module (Table 1), six out of 34 are found in co‐expression module M5, together with most DEGs. This includes the two cartilage‐related genes, *Runx2* and *Col11a2*, which are also among the six genes that are both differentially spliced and differentially expressed. Of these DSGs, *Runx2*, which codes a transcription factor protein, is of particular interest. Not only is it an essential gene for osteoblast differentiation, but it is also acts as an important integrator of the interaction between the BMP pathway and other major signalling pathways like Hedgehog and Wnt (Rahman et al., 2015). Considering that we find changes in expression in genes from these two pathways, it is possible that changes in the splicing and expression of *Runx2* could be partially mediating the changes in these pathways. Given that some DSGs also have functions consistent with the phenotypic effects of the *Eda* haplotype, changes in alternative splicing could be one of the mechanisms by which differences between the *Eda* haplotypes leads to changes in the lateral plate and lateral line phenotypes.

In summary, our results suggest that several major developmental pathways that have been described in other systems to be involved in the development of ectodermal appendages and the lateral line, like Bmp, Wnt, Fgf and Notch, are probably also involved in mediating the phenotypic effects of the *Eda* haplotype in the lateral plates and lateral line. This suggests that the effect of *Eda* on the lateral plate in threespine stickleback is at least in part mediated by conserved developmental pathways involved in the formation of homologous structures in other vertebrates. However, it is important to note that we only examined a single developmental timepoint after the plates had formed in CC and CL individuals, so there may be other genes or pathways that mediate the effects of the *Eda* haplotype at earlier stages of development. Elucidating the direct causal relationships between these pathways will require examination of expression at additional timepoints as well as manipulative experiments.

### 4.3 Possible effect of the *Eda* haplotype in immunity

The *Eda* haplotype includes two other genes, *Tnfsf13b* and *Garp*, both of which are predicted to have immune functions. However, it is not clear whether *Tnfsf13b* and *Garp* are important for freshwater adaptation or if they are simply tightly linked to the *Eda* haplotype. Although neither *Tnfsf13b* nor *Garp* is a DEG or DSG in the skin or the head kidney, these genes could still be differentially expressed or spliced in tissues or developmental timepoints not sampled in our study. Furthermore, there are coding changes between the C and L haplotype in both genes, which could also have phenotypic effects (Colosimo et al., 2005). Even if these two genes do not contribute to differences in immune function, *Eda* itself could still have an effect in immunity. Thus, we looked for an effect of the *Eda* haplotype on immune‐related genes in two tissues important for immunity in teleost fish: the skin, one of the main physical barriers against pathogens, and the head kidney, one of the main leucocyte producing tissues (Smith et al., 2019). In skin, we found an enrichment of DEGs involved in bradykinin signalling (Figure 5), which are pro‐inflammatory molecules (Kaplan et al., 2002; Marceau & Regoli, 2004), as well as scavenger receptors (Figure 5), which are a diverse family of receptors with roles in homeostasis and innate immunity, including identification and clearance of pathogens and inflammatory signalling (Alquraini & El Khoury, 2020; Canton et al., 2013). This suggests a potential for an effect of the *Eda* haplotype on innate immune responses in skin, in particular inflammation, which could be important to deal with the different pathogens in freshwater and marine environments. However, it is also important to note that the some inflammatory signalling proteins, including bradykinins, have been implicated in bone reabsorption (Epsley et al., 2021; Lerner et al., 1987). Thus, it is also possible that these inflammation‐related genes are associated with homeostasis of the lateral plates rather than mediating inflammatory response differences. However, this still does not exclude the possibility than in an immune challenge scenario, the presence of different *Eda* genotypes could lead to differences in inflammatory response. Our literature research also revealed the presence of two DEGs involved in leucocyte function and/or development (*Ets1* and *Laptm4b)* (Dittmer, 2003; Garrett‐Sinha, 2013), and one DSG (*Tbk1)* that is an important integrator of multiple signalling pathways related to immunity, namely pathogen detection, inflammation and immune response (Helgason et al., 2013). Furthermore, *Laptm4b* was found to interact with *Garp*, one of the genes in the *Eda* haplotype, in mammalian cells (Huygens et al., 2015), raising the prospect that *Garp* could be mediating immune changes in skin between marine and freshwater threespine stickleback. Together, these data suggest a potential for an effect of the *Eda* haplotype on inflammation as well as other immune functions in skin.

We also found an effect, albeit small, of the *Eda* haplotype in the main immune tissue in fish, head kidney. In contrast to skin, the effect of the haplotype in head kidney was solely on splicing (Figure 3b). In the *Eda* CC versus LL comparison, we found 14 DSGs, two of which have important immune functions. The *Itgb2* (integrin beta 2) gene, also known as *Lfa‐1* and *Cd177*, is important for the function and migration of T cells, neutrophils and killer cells (Bai et al., 2017; Barber et al., 2004; Kristóf et al., 2013; Ostermann et al., 2002; Walling & Kim, 2018). The *traf3* (TNF receptor‐associated factor 3) gene plays an important role in antiviral innate immune response (Gao et al., 2021; Oganesyan et al., 2006; Tseng et al., 2010) and in the regulation of B and T white cells (Lin et al., 2015; Yi et al., 2015). Interestingly, beyond immune functions, we also found two DSGs with neuronal development annotations (*Cables1* and *Nup98*) (Table S4). It is possible that changes in the splicing of these genes could lead to changes in the innervation of head kidney between *Eda* genotypes, which could have an influence on how this organ reacts to external stimuli.

It is important to keep in mind that the individuals used in this study were healthy. Thus, it is possible that we are missing effects of the *Eda* haplotype that would only manifest during a situation of immune challenge. However, the results we find in healthy individuals already suggest that the *Eda* haplotype has the potential to influence immunity in two important immune organs, skin and head kidney. These results are consistent with a previous study that found evidence that the *Eda* haplotype affected parasite load and the expression of target immune genes in F2 individuals placed in enclosures in the wild (Robertson et al., 2017). However, due to the large blocks of linkage disequilibrium present in F2 crosses, the effect of mutations linked to the *Eda* haplotype could not be excluded in this study. Thus, although there is accumulating evidence that the *Eda* haplotype affects immunity, future follow‐up work, using the crossing design like in our study, and immune challenge experiments like in Robertson et al. (2017) will be required to definitively establish whether the *Eda* haplotype affects immune phenotypes.

### 4.4 Differentially spliced genes are not more pleiotropic than differentially expressed genes in the skin of threespine stickleback

The effect of the *Eda* haplotype on both gene expression and alternative splicing of different genes putatively related to the same functions raises the question of why some genes are differentially expressed while others are differentially spliced. Even though this likely depends on the specifics of each individual gene, alternative splicing is a possible mechanism to avoid the functional constraints of pleiotropic genes by tinkering with the expression of different isoforms rather than expression of the entire gene. Two recent studies have provided some support for this hypothesis. Using tissue specificity as a proxy for pleiotropy, one study found DSGs to be more pleiotropic than DEGs or the rest of the transcriptome, with DEGs showing lower levels of pleiotropy than the rest of the transcriptome (Rogers et al., 2021). Using gene connectivity and the number of associated GO terms as proxies for pleiotropy, the other study found that both DSGs and DEGs tend to be more pleiotropic than non‐DSGs or non‐DEGs respectively (Jacobs & Elmer, 2021). In contrast to these two studies, we found that pleiotropy (measured as gene co‐expression connectivity) in the stickleback skin DSGs does not differ from the rest of the transcriptome and that DEGs are more pleiotropic than both DSGs and the rest of the transcriptome (Figure 4). These mixed results among the studies could result from the different biological contexts of the studies (genes affected by alleles of a single large effect haplotype in stickleback versus distinct freshwater ecotypes in Artic charr, Jacobs & Elmer, 2021 and males and females in bird species, Rogers et al., 2021) and/or from the use of different pleiotropy proxies (gene co‐expression connectivity vs. tissue specificity). In particular, gene co‐expression connectivity, the proxy used in our study and by Jacobs and Elmer (2021), could be biased towards genes that are DEGs, since these genes will be highly co‐expressed with each other, increasing their connectivity value. However, even if that is the case, the GO enrichment analysis suggests the DEGs are involved in pleiotropic developmental pathways, which is in line with the high connectivity value of these genes. Likewise, it is possible that gene co‐expression connectivity is underestimated in DSGs since the analysis only assesses co‐expression patterns at the gene level and not at the isoform level. Thus, genes with isoforms with different co‐expression patterns would have a noisy co‐expression signature at the gene‐level. This idea is supported by the fact that when we atomized one of the skin DSGs that did not belong to any co‐expression module (*Rmnd5b*) into its individual exons and input the exons as ‘genes’ into the co‐expression analysis, the first exon of Rmnd5b, which is differentially spliced between *Eda* genotypes, was part of the same co‐expression network as most DEGs (Table S6). However, despite these limitations of the connectivity proxy, Jacobs and Elmer (2021) did identify a higher gene co‐expression connectivity of DSGs than non‐DSGs in their study. Thus, it is possible that the differences in the results of the three studies might be related to their different biological contexts. This would suggest that connectivity by itself is not a determining factor for the use of differential splicing to mediate phenotypic differences.

Comparisons of the gene expression levels of DEGs and DSGs offer an alternative explanation. Although gene connectivity tends to increase in genes with higher expression levels (Figure S7), DSGs tend to have higher expression levels than the average of the transcriptome or than DEGs, despite having lower connectivity. There is evidence that highly expressed genes evolve more slowly and are under stronger selective constraints, which has been suggested to be associated with the cost of transcription and/or translation (Drummond et al., 2005; Gout et al., 2010). Theoretical models also predict that highly expressed genes are more likely to be pleiotropic (Guillaume & Otto, 2012). Differential splicing could be a good mechanism to modulate the function of these highly expressed genes by changing the expression of alternative isoforms without affecting the expression level of the highly expressed isoform(s). In general, these results suggest that connectivity and expression level might be important factors in determining whether differential expression or alternative splicing is affected in genes mediating phenotypic effects. However, more studies are needed for a more concrete understanding of whether these factors or others tend to determine the use of differential splicing and differential expression, or whether the use of these regulatory mechanisms is mainly context dependent.

## 5 CONCLUSIONS

Knowing the molecular mechanisms and pathways that connect adaptive genes to adaptive phenotypes is an important step towards understanding why particular genes and genetic changes might be used more often during phenotypic evolution (Stern, 2013). In this study, we tackled this question by asking what genes and regulatory mechanisms are differentially affected by the marine and freshwater alleles of the *Eda* haplotype, a locus involved in lateral plate and lateral line differences between marine and freshwater sticklebacks. Our results show that the *Eda* haplotype affects hundreds of genes with different biological functions, like signalling, development and immunity. These include conserved pathways and genes involved in bone formation and neuromast development, suggesting that the effects of the *Eda* haplotype on lateral plates and the patterning of the lateral line are mediated, at least in part, by conserved pathways. We also found that differential expression was not the only regulatory mechanism at play, but that some genes were instead affected by changes in alternative splicing patterns. Furthermore, gene co‐expression connectivity and expression levels were different between these two categories of genes, suggesting that these factors might influence the types of genetic changes that underlie adaptive phenotypic evolution.

## AUTHOR CONTRIBUTIONS

Carlos E. Rodríguez‐Ramírez and Catherine L. Peichel conceived and designed the study; Melanie Hiltbrunner and Verena Saladin performed the experiments; Stephanie Walker contributed to the literature research of immune functions in differentially expressed genes; Carlos E. Rodríguez‐Ramírez analysed and interpreted the data with input from Araxi Urrutia and Catherine L. Peichel. Carlos E. Rodríguez‐Ramírez wrote the paper with input from Catherine L. Peichel.

## CONFLICT OF INTEREST STATEMENT

The authors declare that they have no conflicts of interest regarding this study.

## BENEFIT‐SHARING STATEMENT

Benefits from this research accrue from the sharing of our data and results on public databases as described above.

## Supporting information


Figures S1‐S7

Table S1

Table S2

Table S3

Table S4

Table S5

Table S6

Table S7

Table S8

## Data Availability

Raw RNA‐seq data are available at the NCBI SRA (Bioproject number PRJNA961361), and all scripts used for the analyses are available on Dryad (doi: 10.5061/dryad5x69p8d87).
