# Supplementary material for: Molecular mechanisms of Eda‐mediated adaptation to freshwater in threespine stickleback
Source: Mol Ecol. 2023 May 16;34(15):e16989. doi: 10.1111/mec.16989 (PMC12288785; doi:10.1111/mec.16989)
Supplement: Supplementary file 1 — Figures S1‐S7 [file MEC-34-e16989-s008.pdf]

Supplementary Information for the manuscript *"Molecular mechanisms of Eda-mediated adaptation to freshwater in threespine stickleback"*

Carlos E. Rodríguez-Ramírez[1], Melanie Hiltbrunner[1], Verena Saladin[1], Stephanie Walker[1], Araxi Urrutia[2,3], Catherine L. Peichel[1*]
[1]Division of Evolutionary Ecology, Institute of Ecology and Evolution, University of Bern, Bern 3012, Switzerland
[2]Department of Biology and Biochemistry, and Milner Centre for Evolution, University of Bath, Bath, BA2 7AY, UK
[3]Institute of Ecology, UNAM, 04510, Mexico City, Mexico
**corresponding author, email: catherine.peichel@unibe.ch*

1. Supplementary Figures

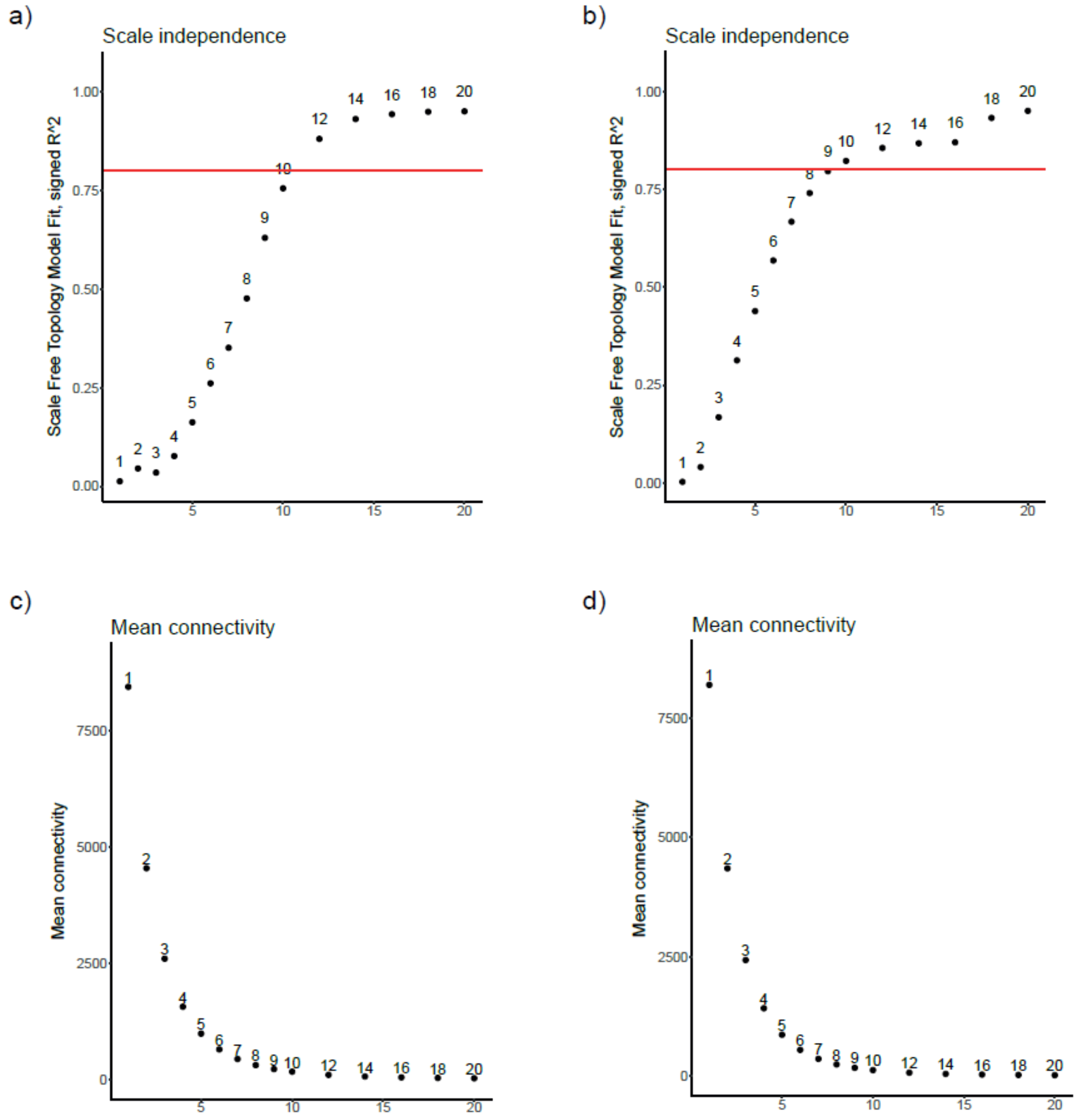

Figure S1. Scale-free topology model fits for **a)** skin and **b)** head kidney, and mean connectivity for **c)** skin and **d)** head kidney for different values of beta for co-expression analysis with WGCNA.

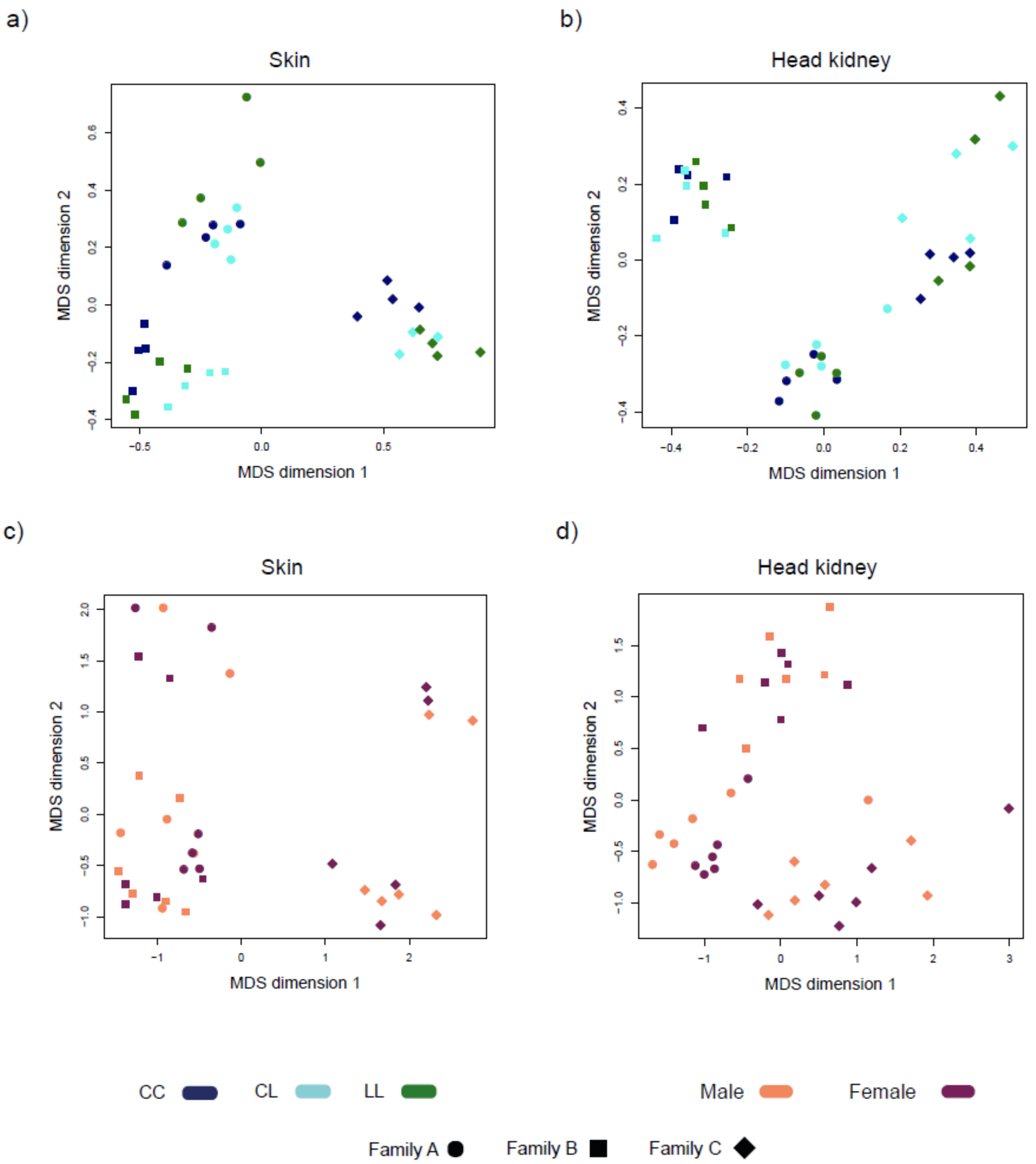

Figure S2. MDS plot of the pairwise distances between the gene expression profiles in skin and head kidney samples. MDS plots include all genes in the **a)** skin and **b)** head kidney transcriptomes or the top pairwise 500 genes with greatest expression fold-changes between each pair of samples in the **c)** skin and **d)** head kidney transcriptomes.

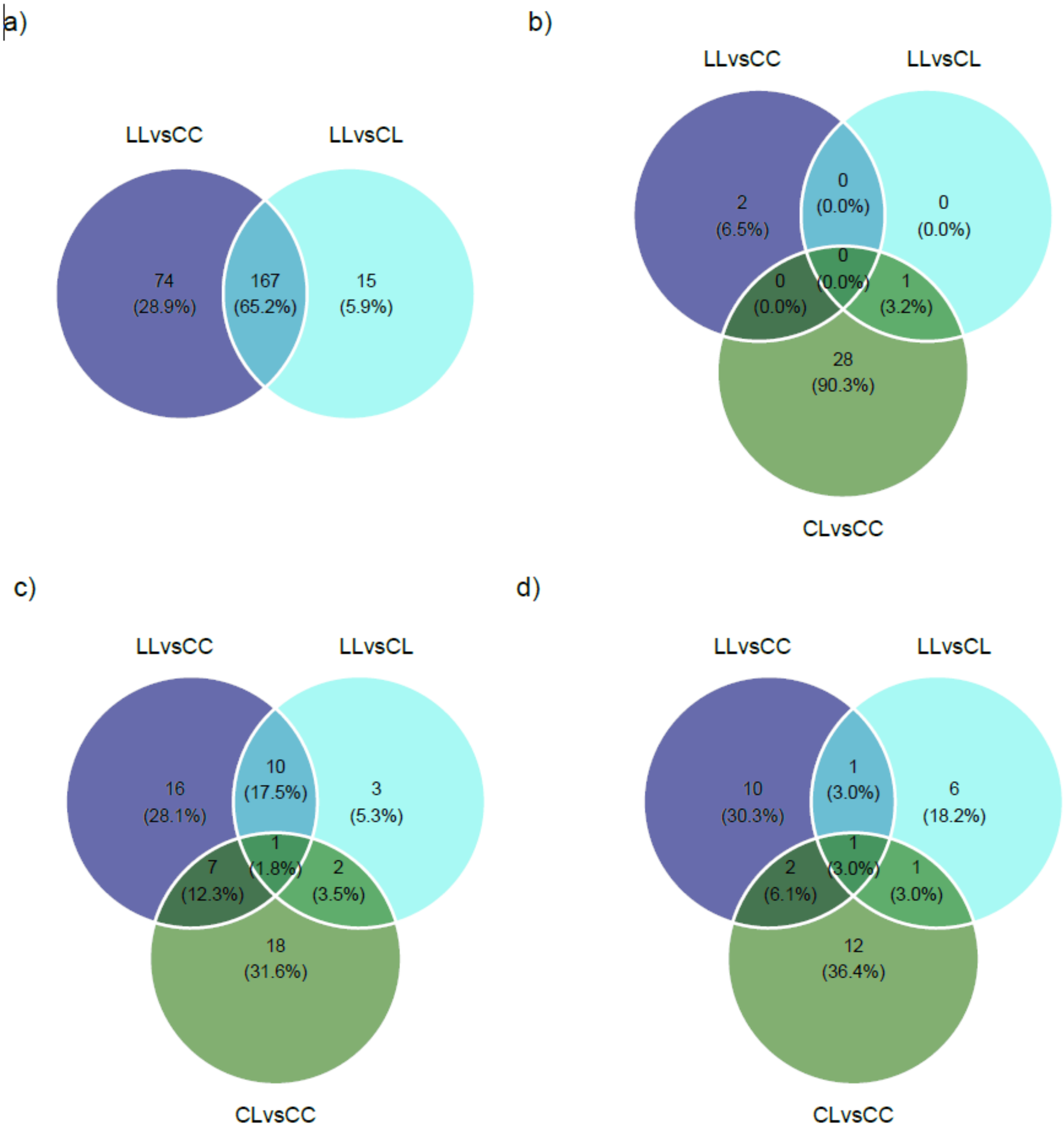

Figure S3. Overlap of genes identified in the different *Eda* genotype pairwise comparisons. **a)** DEGs in skin, **b)** DEGs in head kidney, **c)** DSGs in skin, and **d)** DSGs in head kidney.

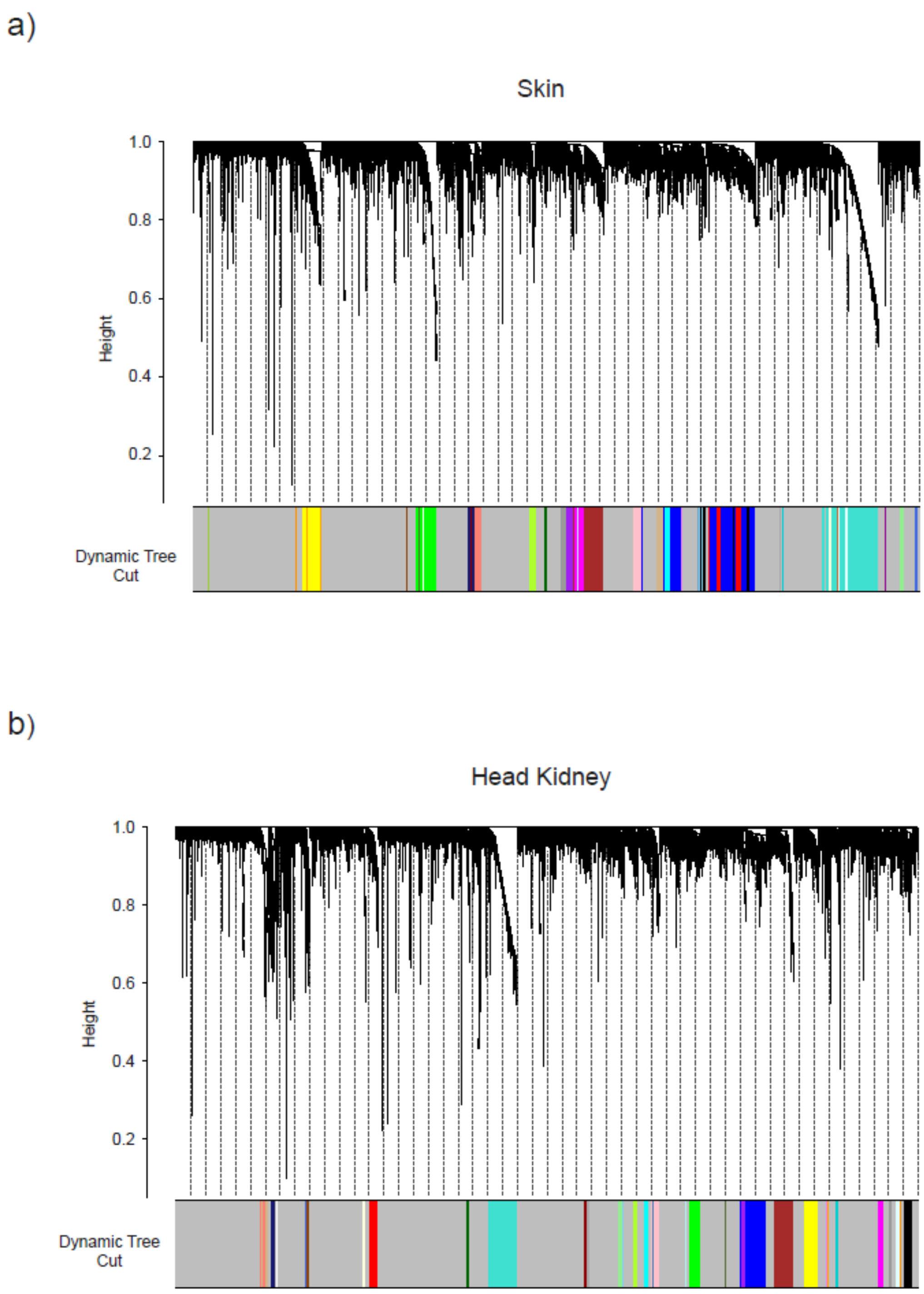

Figure S4. Topology Overlap Matrix (TOM) dissimilarity dendrograms calculated by WGCNA for beta = 14, for **a)** skin and **b)** head kidney. Each vertical line on the dendrogram represents a gene. Dynamic Tree Cut graph underneath represents identified modules. See Supplementary Table S8 for module ID and colour guide.

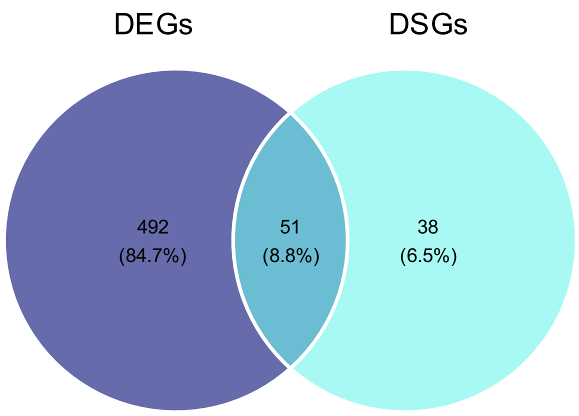

Figure S5. Overlap between the Gene Ontology (GO) Terms between DEGs and DSGs of the skin *Eda* LL vs CC comparison.

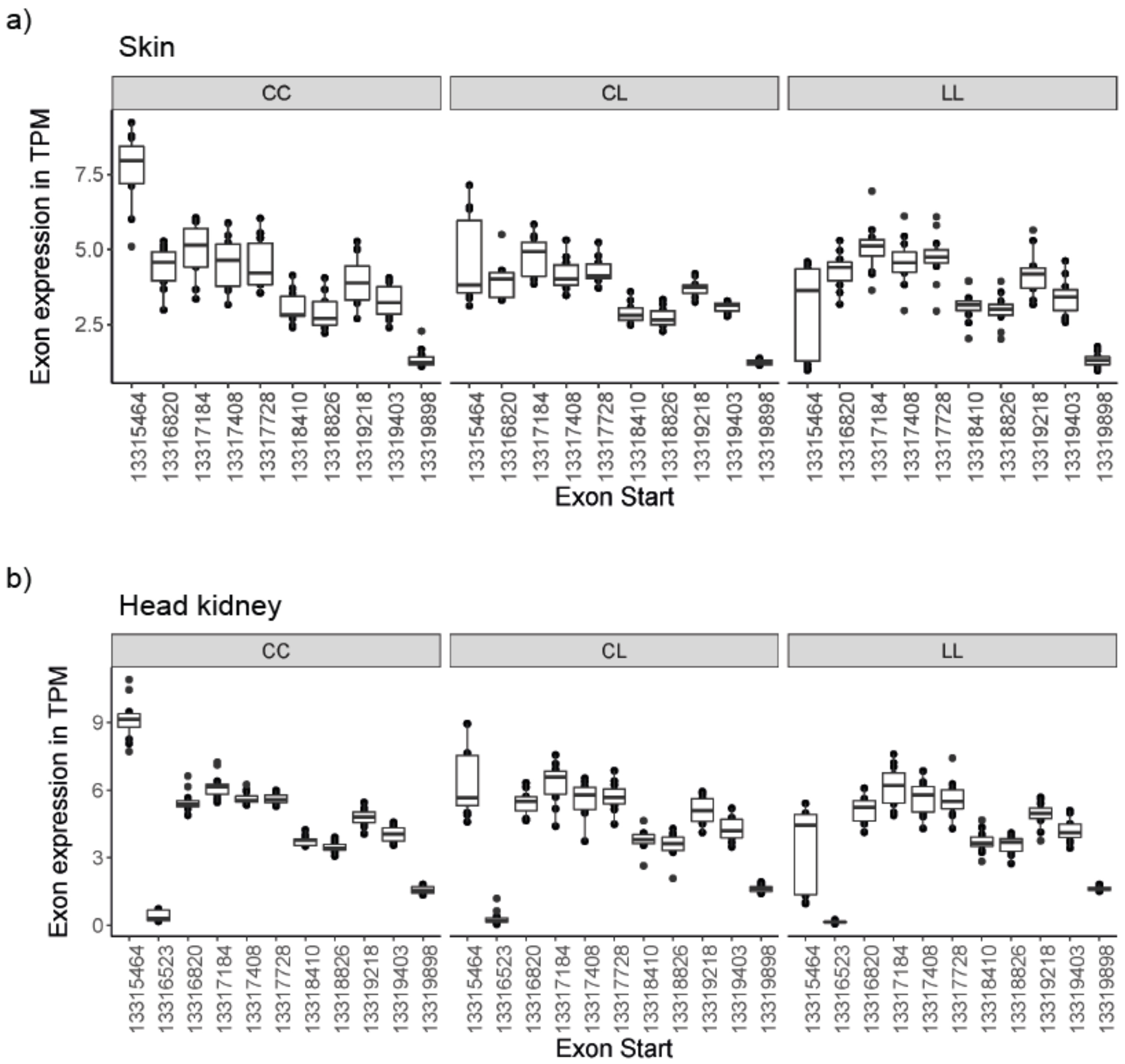

Figure S6. Exon expression plots for *Rmnd5b* in **a)** skin and **b)** head kidney of CC, CL and LL individuals. *Rmnd5b* first exon (starting at position 13315464 of chromosome IV), is differentially used between genotypes. The second exon (starting at position 13316523) is not represented in the skin exon plots because it was too lowly expressed to be included in the analysis.

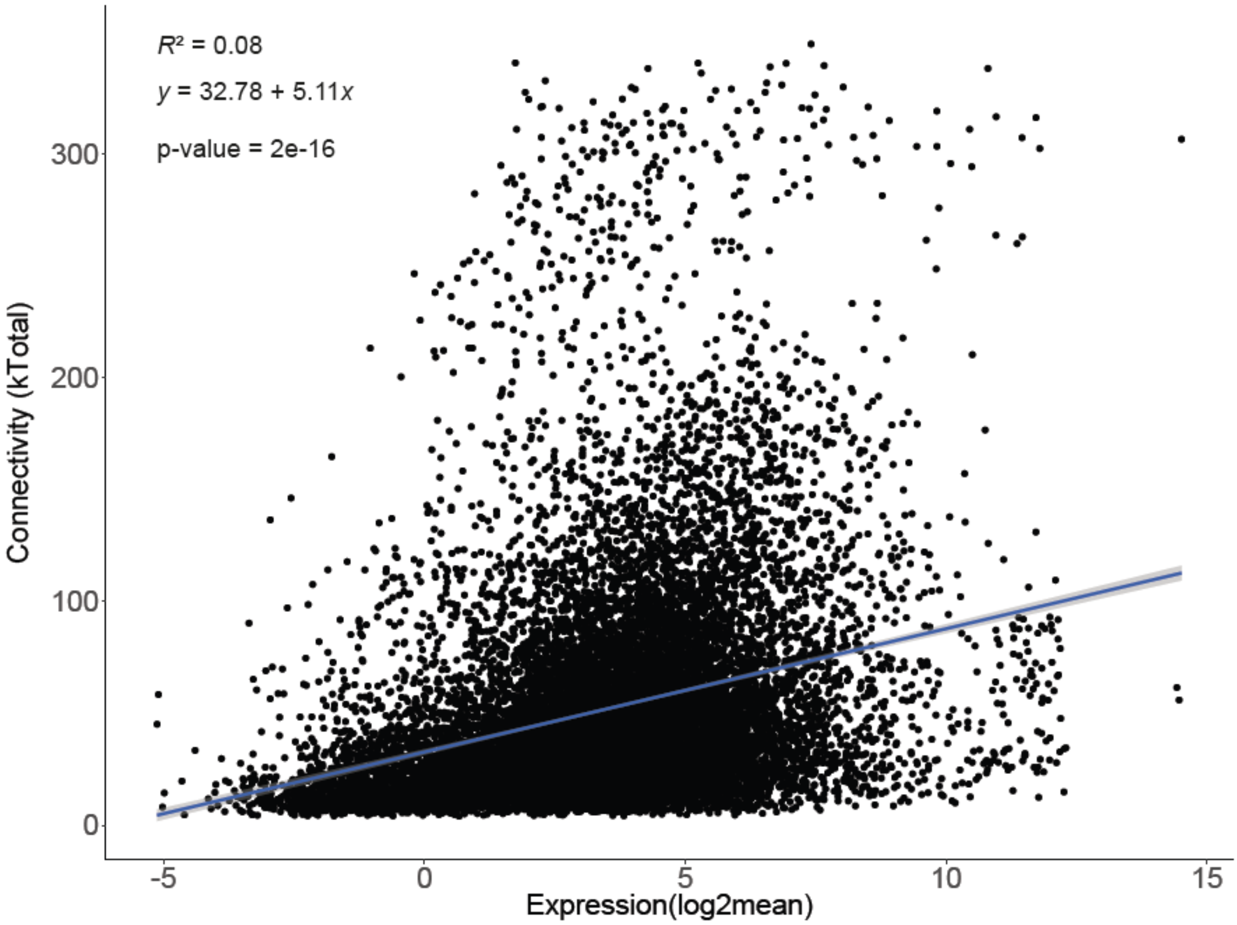

Figure S7. Scatterplot of the positive relationship between connectivity and gene expression across the skin transcriptome.

2. Supplementary Tables

Table S1. Markers used to genotype the parents and F3 individuals used for this study.

Table S2. (A) Differentially expressed genes (DEGs) and (B) differentially spliced genes (DSGs) identified across the three pairwise *Eda* genotype comparisons in skin and head kidney.

Table S3. GO enrichment results for (A) skin DEGs and (B) gene co-expression modules M5 and M27 (in skin) and M3 (in head kidney).

Table S4. GO Terms present in (A) DEGs and (B) DSGs of the *Eda* skin LL vs CC comparison, and (C) skin gene co-expression module M27

Table S5. DEGs and DSGs without immune GO Terms but with immune functions found in the literature.

Table S6. Gene co-expression module and connectivity of all genes in the (A) skin and (B) head kidney transcriptomes, and in the same transcriptomes when separating *Rmnd5b* into its individual exons in (C) skin and (D) head kidney.

Table S7. SNPs in the RNAseq reads mapping to *Rmndb5b*, their genotype counts in CC and LL individuals (ref, alt or het) and genotype mismatches between CC and LL individuals.

Table S8. WGCNA module colour table for WGCNA dendrograms for (A) skin and (B) head kidney in Figure S4.
